# A 15 Year Ecological Comparison for the Market Dynamics of Minnesota Community Pharmacies from 2002 to 2017

**DOI:** 10.3390/pharmacy6020050

**Published:** 2018-06-02

**Authors:** Anthony W. Olson, Jon C. Schommer, Ronald S. Hadsall

**Affiliations:** College of Pharmacy, University of Minnesota, 308 Harvard Street, S.E., Minneapolis, MN 55455, USA; schom010@umn.edu (J.C.S.); hadsa001@umn.edu (R.S.H.)

**Keywords:** community pharmacy, market dynamics, independent community pharmacy, chain community pharmacy, Minnesota

## Abstract

**Background:** Understanding the factors that influence the market entry, exit, and stability of community pharmacies (i.e., market dynamics) is important for stakeholders ranging from patients to health policymakers and small business owners to large corporate institutions. **Objective:** The study’s first objective was to describe the market dynamics of community pharmacies for Minnesota counties in 2002, 2007, 2012, and 2017 by associating county (a) population density and (b) metropolitan designation with the change in the number of ‘All community pharmacies,’ ‘Chain community pharmacies’, and ‘Independent community pharmacies’. The study’s second objective was to describe the number and proportion of community pharmacies for Minnesota counties in 2002, 2007, 2012, and 2017 by (1) ‘Business Organization Structure’ and (2) ‘Pharmacy Type.’ **Methods:** County-level data were obtained from the Minnesota Board of Pharmacy, US Census Bureau, and Minnesota State Demographic Center for 2002, 2007, 2012, and 2017. Findings were summarized and the associations between study variables described using descriptive statistics. **Results:** The ratio of ‘Independent community pharmacies’ to ‘Chain community pharmacies’ was about 1:1 (466:530) in 2002, 1:2 (352:718) in 2007, 1:2 (387:707) in 2012, and 1:3 (256:807) in 2017. There was not a consistent relationship that carried through the 15 year analysis between county population density and metropolitan designation and the market dynamic patterns of community pharmacies. The types of pharmacy in Minnesota changed significantly over the study with increases in state, regional, and national chains and declines in single entity and small chain independents. There were also notable declines in mass merchandiser community pharmacies and increases in clinic and medical center community pharmacies. **Discussion:** The findings suggest that different or additional factors beyond traditional market dynamic predictors of population density and metropolitan designation were at play in each five year interval of this study. We propose that the traditional dichotomy of independent and chain community pharmacy groupings no longer provide an optimal characterization for the market dynamics of pharmacies today. Instead, community pharmacies may be better organized by their capacity to operate as healthcare access points that provide and are reimbursed for patient care and public health services like medication therapy management, immunizations, and more. **Conclusions:** The findings showed that community pharmacy distribution in Minnesota’s 87 counties has shifted between 2002 and 2017 from traditional retail models to emerging healthcare models based on population health needs. This signals the need for not only a new approach for tracking community pharmacy market dynamics but also adjustments by community pharmacies to remain relevant in a new environment of patient care services.

## 1. Introduction

### 1.1. Background

Over half of actively practicing pharmacists work in a community pharmacy, making the market dynamics of this practice setting very impactful on the pharmacist labor market [1,2]. Community pharmacies in the United States are an important access point for healthcare products and services involving medication, disease management, and public health [3,4,5,6]. An understanding of the factors that influence the market entry, exit, and stability of community pharmacies (i.e., market dynamics) of all types (e.g., mass merchandiser, clinic, etc.) is important for stakeholders ranging from patients to health policymakers and small business owners to large corporate institutions [7].

Previous research by Schommer et al. has demonstrated that market entry, exit, and geographical distribution of community pharmacies (i.e., market dynamics) on the county level in Minnesota between 1992 and 2012 could be explained using environmental attributes drawn from organizational behavior theory [8,9]. This approach suggests that environmental factors like population density and a county’s designation as a rural or metropolitan area affect the business decisions made by community pharmacies, which influences access to community pharmacy services. The results of this research found that county characteristics like a metropolitan designation and growth in population density were significantly associated with gains in the overall number of community pharmacies, but relative declines in the number of independent community pharmacies. The increases in chain community pharmacies and all community pharmacies overall was attributed to metropolitan counties possessing the infrastructure (e.g., transportation, communication, health systems), workforce pool (i.e., source of pharmacists to fulfill staffing requirements), and market demands for items other than medications. Meanwhile, independent community pharmacies declined while chain community pharmacies grew in these counties potentially because the former were unable to capitalize on these resources to the same extent as the latter. In counties with low or negative population density growth, the number independent community pharmacies declined likely due to insufficient demands in the marketplace for sustainably operating a pharmacy as generally there was no chain community pharmacy growth in these areas. Schommer et al.’s research also documented changes in the face of community pharmacies in Minnesota, with the gradual rise of community pharmacies in mass merchandiser stores, regional supermarkets, and health care clinics/medical centers amongst declines in traditional retail pharmacy.

Since the publication of this research, the role, and importance of community pharmacies has changed and grown in terms of providing public health (e.g., immunizations, opioid rescue drugs, drug disposal) and population health services (medication management, health screening, specialty pharmacy) [3,10,11,12,13,14,15,16,17]. Other changes like the recovery from the economic downturn of 2008, healthcare legislation (e.g., Patient Protection and Affordable Care Act, Health Care and Education Reconciliation Act), the growth of continuity of care models, and the rise of outcome-based reimbursement systems warrant continued investigation of the market dynamics of community pharmacies in Minnesota [18,19,20].

### 1.2. Objectives

The first objective of this study was to describe the market dynamics of community pharmacies for each county in the state of Minnesota every five years over a 15 year period (i.e., 2002–2017). The development of methods for fulfilling this objective was guided by previous work done by Schommer et al., which associated county (a) population density and (b) metropolitan designation with the change in the number of ‘All community pharmacies,’ ‘Chain community pharmacies,’ and ‘Independent community pharmacies [9]’.

The second objective was to describe the number and proportion of community pharmacies for each county in the state of Minnesota every five years over a 15 year period (i.e., 2002–2017) by (1) ‘Business Organization Structure’ and (2) ‘Pharmacy Type.’ These two variables reflect varying approaches to generating revenue and providing health services by community pharmacies in response to environmental factors (i.e., market dynamics).

## 2. Methods

### 2.1. Data Sources

County-level data for the number, names, and locations of community pharmacies in Minnesota was obtained from the Minnesota Board of Pharmacy for 2002, 2007, 2012, and 2017 [21]. Population density and metropolitan designation information for each county were obtained from the US Census Bureau and Minnesota State Demographic Center [22,23].

### 2.2. Data Analysis

Each pharmacy in Minnesota was categorized from state licensing board records by their location (i.e., county), ‘Business Organization Structure,’ and ‘Pharmacy Type.’ Descriptive statistics were used for tabulating and summarizing the findings for the years 2002, 2007, 2012, and 2017. Temporal associations between independent and dependent variables were described using Pearson Chi-square analysis given there were only 87 cases (i.e., counties). A *p*-value of less than 0.05 was the significance threshold used for each test and was computed using IBM SPSS version 24.0 at the University of Minnesota, College of Pharmacy. Additional statistical computations for likelihood-ratio and fisher’s exact tests were also run for each association to detect any findings unstable and affected by cell size. *p*-values for all study comparisons were found to be stable, nearly identical, and therefore none altering the interpretation of significance. 

### 2.3. Study Objective 1

#### 2.3.1. Dependent Variables

Changes in frequency for three dependent variables were used to fulfill study objective 1: ‘All Community Pharmacies,’ ‘Independent community pharmacies’, and ‘Chain community pharmacies’. There were operationally defined as:

- All community pharmacies: Per the state of Minnesota, an “established place(s) in which prescriptions, drugs, medicines, chemicals, and poisons are prepared, compounded, dispensed, vended, distributed, or sold to or for the use of non-hospitalized patients and from which related pharmaceutical care services are provided [24]”.
- Independent community pharmacies: A community pharmacy owned as a single entity or as part of an organization comprising of 10 or fewer community pharmacies.
- Chain community pharmacies: Any community pharmacy owned as part of an organization comprising of more than 10 community pharmacies.

Frequency changes for each dependent variable were totaled and tracked for each Minnesota county in 2002, 2007, 2012, and 2017. Each of these variables was coded as: −1 if the county lost pharmacies, 0 if the number of pharmacies in county stayed the same, and 1 if the county gained pharmacies. 

#### 2.3.2. Independent Variables

‘Change in Population Density’ was defined as the change in person per square mile in each county for every five years from 2002–2017. This variable was coded as: −1 = negative change, 0 = change was from 0 to 5 people per square mile, and 1 = change was greater than 5 people per square mile. This variable represented the change in population for each county in a standardized unit of measurement. Metropolitan designation was defined by the US Census Bureau (core urban area of 50,000 or more population) wherein counties were coded as 0 = non-metro area and 1 = metro area. 

### 2.4. Study Objective 2

#### Variables

Two variables were used to study the second objective: (1) ‘Business Organization Structure’ and (2) ‘Pharmacy Type.’ ‘Business Organization Structure’ related to the number of pharmacies under common ownership and the size of the organization’s geographic markets. It was operationally defined as:

- Single entity: A business organization comprised of one pharmacy in a local market that would be classified under ‘Independent community pharmacies’ for objective 1.
- Small chain: A business organization comprised of 2–10 community pharmacies under common ownership (typically located in a local market) that would be classified under ‘Independent community pharmacies’ for objective 1.
- State/regional chain: A business organization comprised of greater than 10 community pharmacies under common ownership; distributed throughout Minnesota or the Midwest Region (Iowa, Illinois, Indiana, Kansas, Michigan, Minnesota, Missouri, North Dakota, Nebraska, Ohio, South Dakota, Wisconsin) and that would be classified under ‘Chain community pharmacies’ for objective 1.
- National chain: greater than 10 community pharmacies under common ownership; typically comprised of more than 1000 community pharmacies nationwide, located in most of the 50 states, and that would be classified under ‘Chain community pharmacies’ for objective 1.

The variable of ‘Pharmacy Type’ relates to the square footage devoted to the pharmacy department, the proportion of the business’ revenue coming from the pharmacy department, and the typical reason for patronizing the business [1,2]. It was operationally defined as:

- Health & Personal Care: establishment is considered a pharmacy that also has a “front end”. A relatively large amount of square footage is devoted to the pharmacy and over-the-counter products. Revenue from the pharmacy and over-the-counter product sales is relatively large. The typical reason for patronizing the business is to “go to the pharmacy.” Locational convenience is a primary patronage motive. This type of pharmacy has also been known as a retail pharmacy.
- Mass merchandiser: establishment is considered a big box retail store that also has a “pharmacy.” A relatively small amount of square footage is devoted to the pharmacy and over-the-counter products. Revenue from the pharmacy and over-the-counter product sales is relatively small. The typical reason for patrons to visit the business is to “go to the big box retailer.” Retail shopping convenience is a primary patronage motive.
- Supermarket: establishment is considered a grocery store that also has a “pharmacy.” A relatively small amount of square footage is devoted to the pharmacy and over-the-counter products. Revenue from the pharmacy and over-the-counter product sales is relatively small. The typical reason for patronizing the business is to “go to the grocery store.” Grocery shopping convenience is a primary patronage motive.
- Clinic/medical center: establishment is considered a clinic that also has a “pharmacy”. A relatively small amount of square footage is devoted to the pharmacy and over-the-counter products. Revenue from the pharmacy and over-the-counter product sales is relatively small. The typical reason for patronizing the business is to “go to the clinic”. In some cases, the pharmacy is a stand-alone business but is still considered to be closely associated with the clinic or medical center that is nearby. In many cases, the pharmacy name is the same as the clinic name (XYZ Clinic, XYZ Medical Center, XYZ Pharmacy). Health care visit convenience is a primary patronage motive.
- Specialty: establishment is considered a specialty business. Typically, all of the square footage is devoted to the pharmacy. Revenue for this business typically comes completely from the specialty services offered by the pharmacy. The typical reason for patronizing the business is to “receive unique pharmaceutical services” to meet patient care needs. Examples of specialty pharmacies include those focused upon renal services, compounding, veterinary pharmacy, long-term care, oncology, infusion, nuclear, outpatient treatment centers, HIV medication services, specialty pharmaceuticals. Need for specialty services is a primary patronage motive.

## 3. Results

**Figure A1:**
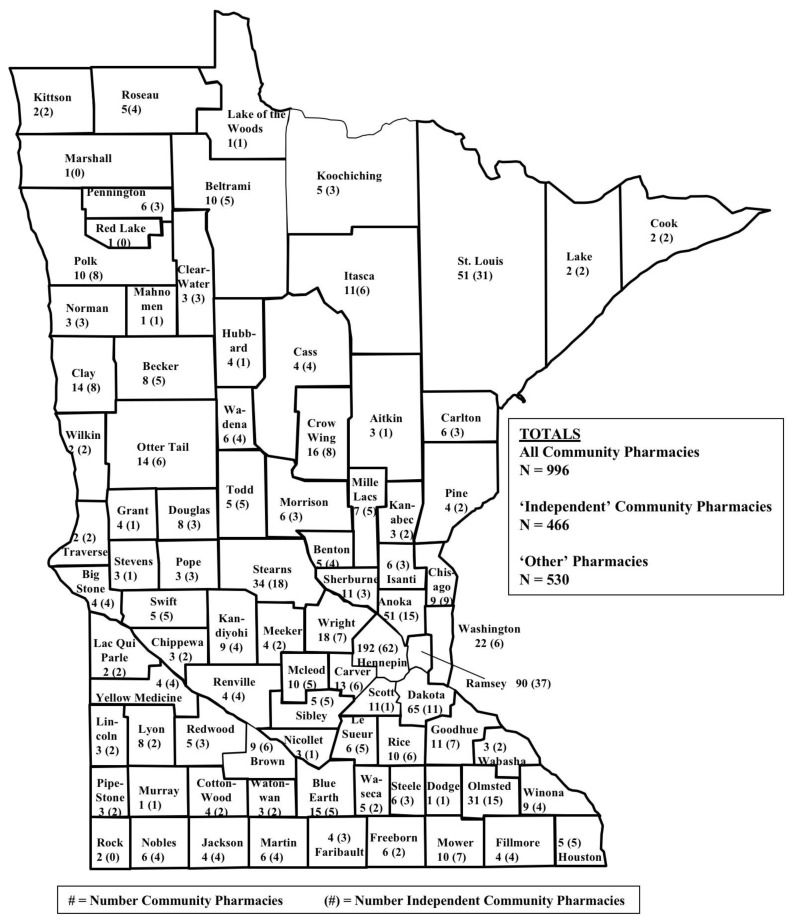
Community pharmacies in Minnesota counties (2002) [9].

**Figure A2:**
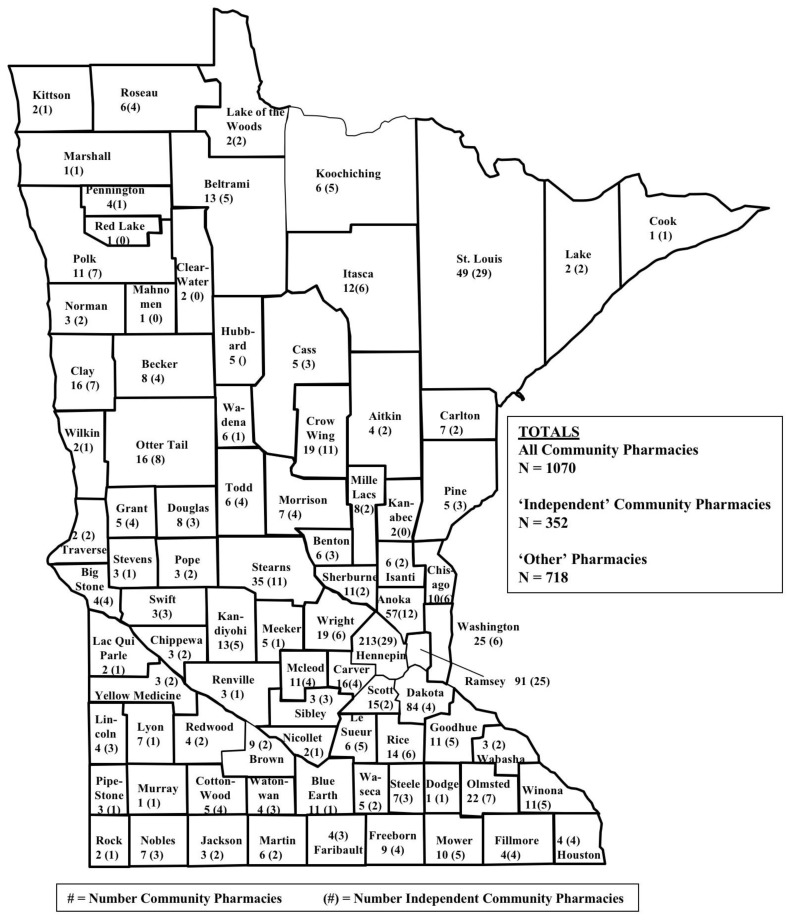
Community pharmacies in Minnesota counties (2007).

**Figure A3:**
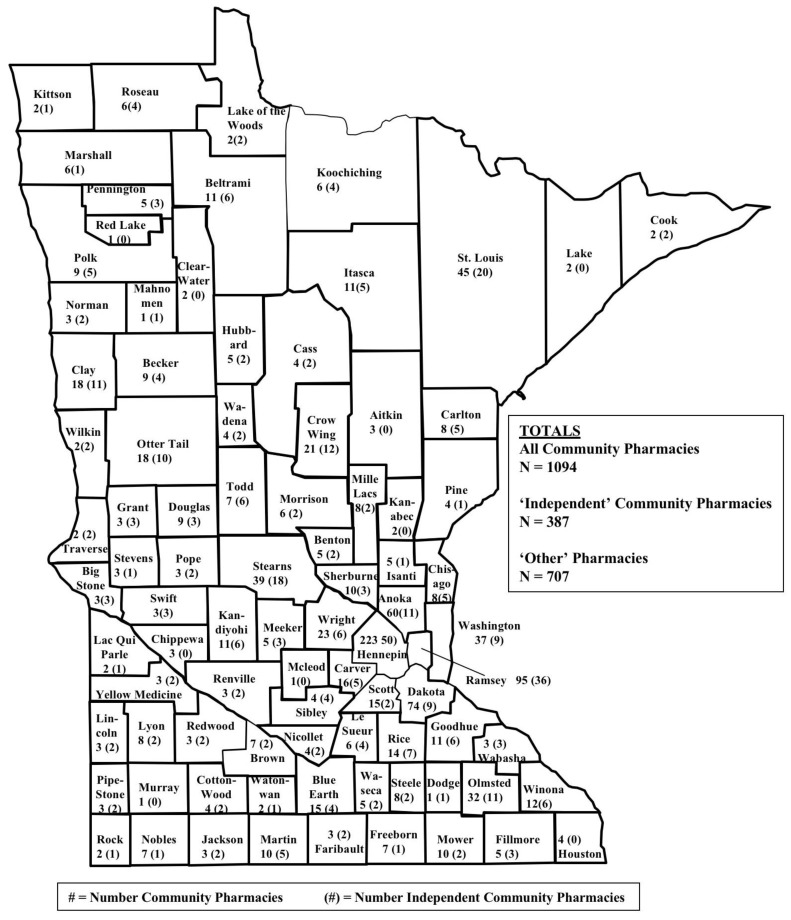
Community pharmacies in Minnesota counties (2012) [9].

**Figure A4:**
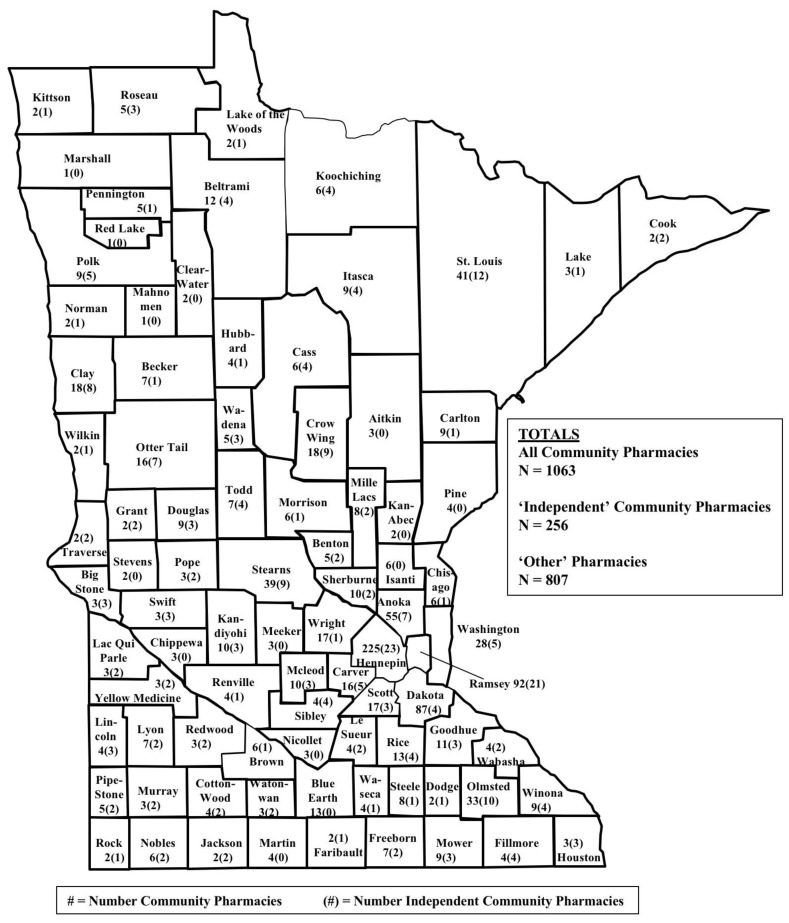
Community pharmacies in Minnesota counties (2017).

The first objective of this study was to describe the market dynamics of community pharmacies for each county in the state of Minnesota every five years over a 15 year period (i.e., 2002–2017). Figure A1, Figure A2, Figure A3 and Figure A4 in the Appendix A depict the county totals of ‘All community pharmacies,’ ‘Independent community pharmacies’, and ‘Chain community pharmacies’ in Minnesota for 2002, 2007, 2012, and 2017. The results show that every county in Minnesota had at least one community pharmacy for all years which data were collected, but only four counties had only one community pharmacy throughout the study. The total number of community pharmacies in Minnesota grew by 7 percentage points between 2002 and 2007, grew by 2 percentage points between 2007 and 2012, and fell by 3 percentage points between 2012 and 2017. The number of ‘Independent community pharmacies’ in Minnesota fell by 25 percentage points between 2002 and 2007, grew by 10 percentage points between 2007 and 2012, and fell by 33 percentage points between 2012 and 2017. The ratio of ‘Independent community pharmacies’ to ‘Chain community pharmacies’ registered roughly 1:1 (466:530) in 2002, 1:2 (352:718) in 2007, 1:2 (387:707) in 2012, and 1:3 (256:807) in 2017.

The market dynamics for Minnesota’s 87 counties for each five year interval between 2002 and 2017 are shown in Table 1. The proportion of Minnesota’s counties that gained community pharmacies in each five year interval declined over time (46% for 2002–2007, 29% for 2007–2012, and 22% for 2012–2017). The proportion of Minnesota’s counties that lost community pharmacies in each five year interval increased over time (18% for 2002–2007, 29% for 2007–2012, and 38% for 2012–2017). The proportion of Minnesota’s counties that maintained the same number of community pharmacies in each five year interval remained fairly stable over time (36% for 2002–2007, 43% for 2007–2012, and 40% for 2012–2017). The five year interval for 2007–2012 stood in contrast to the time periods before and after it, with opposing effects on ‘Independent community pharmacies’ and ‘Chain community pharmacies.’ During this time period, ‘Independent community pharmacies’ in Minnesota counties showed greater gains (38% in 2007–2012 vs. 20% in 2002–2007 & 16% in 2012–2017) and fewer losses (33% in 2007–2012 vs. 55% in 2002–2007 & 54% in 2012–2017). Meanwhile, ‘Chain community pharmacies’ in Minnesota counties showed greater losses (31% in 2007–2012 vs. 8% in 2002–2007 & 16% in 2012–2017) and fewer gains (31% in 2007–2012 vs. 56% in 2002–2007 & 39% in 2012–2017).

**Figure 1:**
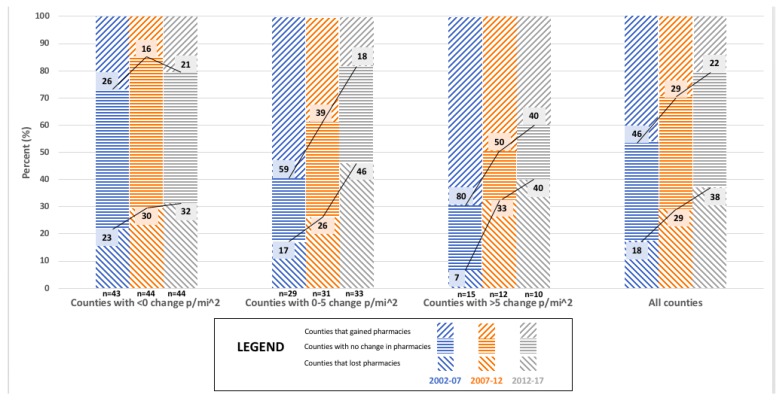
The relationship between change in population density (p/mi^2^) and market dynamics for ‘All community pharmacies’ in Minnesota counties for 2002, 2007, 2012, and 2017 (N = 87).

**Figure 2:**
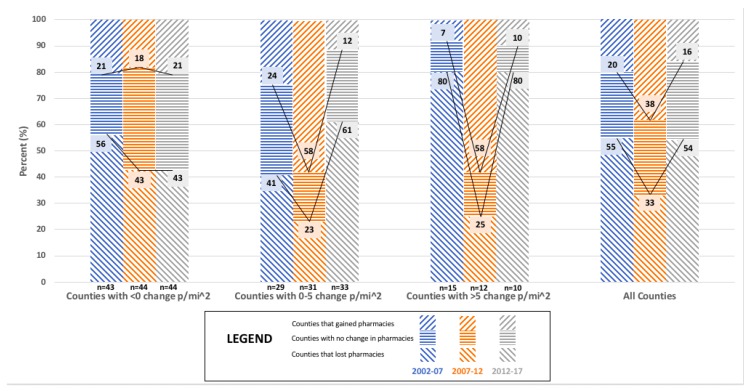
The relationship between change in population density (p/mi^2^) and market dynamics for ‘Independent community pharmacies’ in Minnesota counties for 2002, 2007, 2012, and 2017 (N = 87).

**Figure 3:**
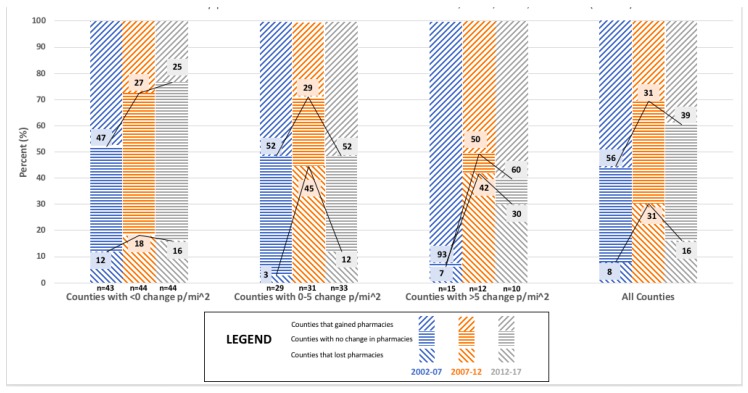
The relationship between change in population density (p/mi^2^) and market dynamics for ‘Chain community pharmacies’ in Minnesota counties for 2002, 2007, 2012, and 2017 (N = 87).

Figure 1, Figure 2 and Figure 3 present findings that relate the change in population density and market dynamics for each five year interval between 2002 and 2017 for the 87 counties of Minnesota. Overall, counties with greater growth in population density lost fewer and gained more community pharmacies than counties with less growth in population density. This association between population density and the change in the total number of community pharmacies can be found in Figure 1 and was statistically significant for 2002–2007 (*p* = 0.002; See Appendix A Table A1), but not 2007–2012 (*p* = 0.052; See Appendix A Table A2) and 2012–2017 (*p* = 0.349; See Appendix A Table A3). 

The relationship between population density with the number of ‘Independent community pharmacies’ is shown in Figure 2, which varied in direction and significance between the five year intervals. For 2002–2007, more than half of counties lost ‘Independent community pharmacies.’ For 2007–2012, the greatest proportional increases for ‘Independent community pharmacies’ occurred in counties that grew in population density. For 2012–2017, more than half of Minnesota counties lost pharmacies with the proportional losses positively relating to greater levels of population density. The association between population density and change in the number of ‘Independent community pharmacies’ was statistically significant for 2007–2012 (*p* = 0.005; See Appendix A Table A2), but not for 2002–2007 (*p* = 0.185; See Appendix A Table A1) and 2012–2017 (*p* = 0.234; See Appendix A Table A3). 

Figure 3 shows the relationship between population density with the number of ‘Chain community pharmacies’ varied in direction and significance between the five year intervals. For 2002–2007, almost 50% of counties in each population density category gained ‘Chain community pharmacies,’ with no more than 12% losing ‘Chain community pharmacies’. For 2007–2012, almost a third of all Minnesota counties lost ‘Chain community pharmacies’ with the greatest proportional declines occurring in counties that grew in population density. However, counties with population density growth greater than 5 persons per square mile gained more ‘Chain community pharmacies’ than counties with less growth or decline in population density. The proportion of counties with large increases in population density lost as many pharmacies as were gained (31% lost vs. 31% gained). For 2012–2017, a little over 20% of all Minnesota counties gained pharmacies with most of the proportional increases occurring in counties that grew in population density. A similar proportion of counties lost pharmacies across the three population density groupings (<0 p/mi^2^: 32%, 0–5 p/mi^2^: 46%, >5 p/mi^2^: 40%). The association between population density and the change in the number of ‘Chain community pharmacies’ was statistically significant for 2002–2007 (*p* = 0.014; See Appendix A Table A1), 2007–2012 (*p* = 0.009; See Appendix A Table A2) and 2012–2017 (*p* = 0.022; See Appendix A Table A3).

**Figure 4:**
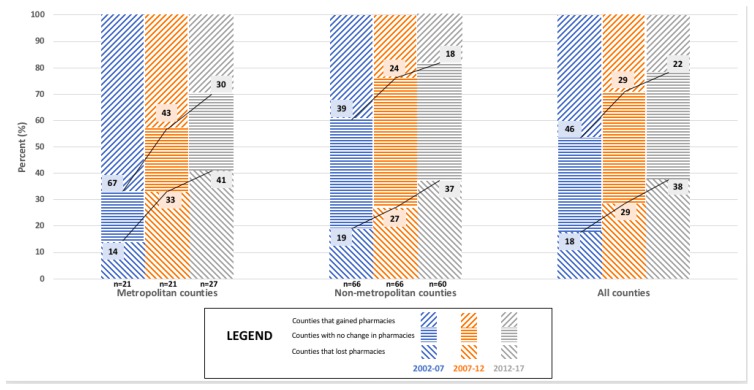
The relationship between Metropolitan Designation and market dynamics for ‘All community pharmacies’ in Minnesota counties for 2002, 2007, 2012, and 2017 (N = 87).

**Figure 5:**
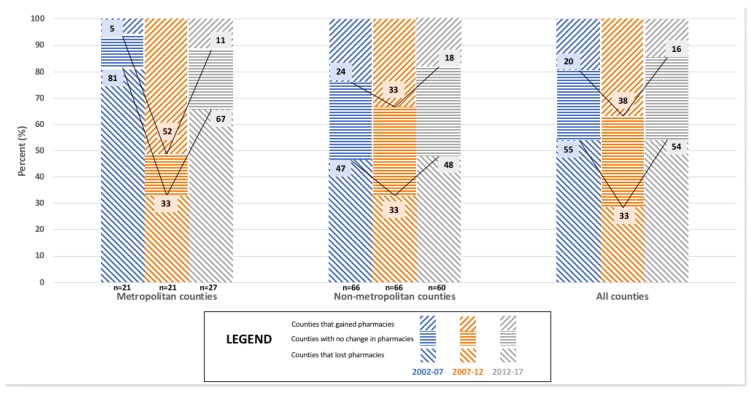
The relationship between Metropolitan Designation and market dynamics for ‘Independent community pharmacies’ in Minnesota counties for 2002, 2007, 2012, and 2017 (N = *87*).

**Figure 6:**
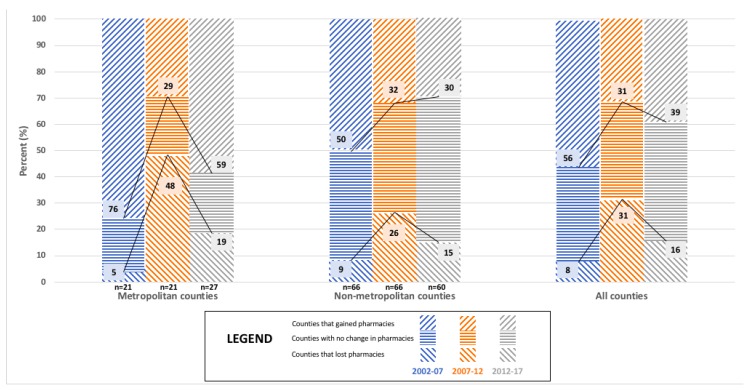
The relationship between Metropolitan Designation and market dynamics for ‘Chain community pharmacies’ in Minnesota counties for 2002, 2007, 2012, and 2017 (N = 87).

Figure 4, Figure 5 and Figure 6 present findings that relate metropolitan designation and market dynamics for each five year interval between 2002 and 2017 for the 87 counties of Minnesota. Metropolitan designation of Minnesota counties was not shown to significantly associate with the change in the total number of community pharmacies for any of the time periods analyzed, which are depicted in Figure 4. However, metropolitan designation of Minnesota counties was shown to significantly associate with the change in the number of ‘Chain community pharmacies’ for 2012–2017 (*p* = 0.013; See Appendix A Table A6), but not for 2002–2007 (*p* = 0.108; See Appendix A Table A4) and 2007–2012 (*p* = 0.138; See Appendix A Table A5) as displayed in Figure 5. Additionally, metropolitan designation of Minnesota counties was shown to significantly associate with the change in the number of ‘Independent community pharmacies’ for 2002–2007 (*p* = 0.021; See Appendix A Table A4), but not for 2007–2012 (*p* = 0.171; See Appendix A Table A5) and 2012–2017 (*p* = 0.282; See Appendix A Table A6) as shown in Figure 6. Results from the 2012–2017 time interval show that almost one third (30%) of counties with a metropolitan designation gained pharmacies compared to less than one fifth (18%) of counties without a metropolitan designation doing the same. A fairly equivalent proportion of both types of counties lost pharmacies (Metro: 41%, Non-Metro: 37%) over the same time period. 

The second objective was to describe the number and proportion of community pharmacies for each county in the state of Minnesota every five years over a 15 year period from 2002 through 2017 by (1) ‘Business Organization Structure’ and (2) ‘Pharmacy Type.’

**Figure 7:**
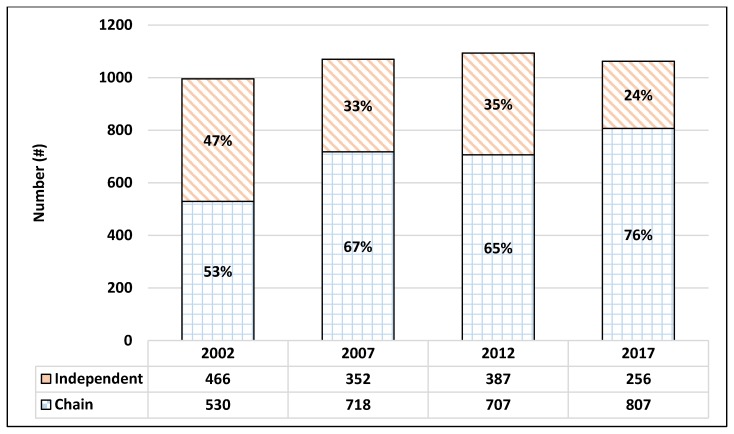
The number of ‘Independent community pharmacies’ and ‘Chain community pharmacies’ in Minnesota for 2002, 2007, 2012, and 2017.

Table 2 shows a summary of Minnesota’s pharmacies by ‘Business Organization Structures’ and ‘Pharmacy Type’ for 2002, 2007, 2012, and 2017. The number of community pharmacies in Minnesota was 996 in 2002, 1070 in 2007, 1094 in 2012, and 1063 in 2017. ‘Independent community pharmacies’ (i.e., Single entity + Small chain) made up 47% (N = 466) of ‘All community pharmacies’ in 2002, with the remaining 53% (N = 530) classified as ‘Chain community pharmacies’ (i.e., State/regional chain + National chain). In 2007, ‘Independent community pharmacies’ made up 33% (N = 352) of ‘All community pharmacies,’ with the remaining 67% (N = 718) classified as ‘Chain community pharmacies.’ In 2012, ‘Independent community pharmacies’ made up 35% (N = 387) of ‘All community pharmacies,’ with the remaining 65% (707) classified as ‘Chain community pharmacies.’ By 2017, ‘Independent community pharmacies’ made up only 24% (N = 256) of ‘All community pharmacies,’ with the remaining 76% (N = 807) classified as ‘Chain community pharmacies’ (see Figure 7). 

**Figure 8:**
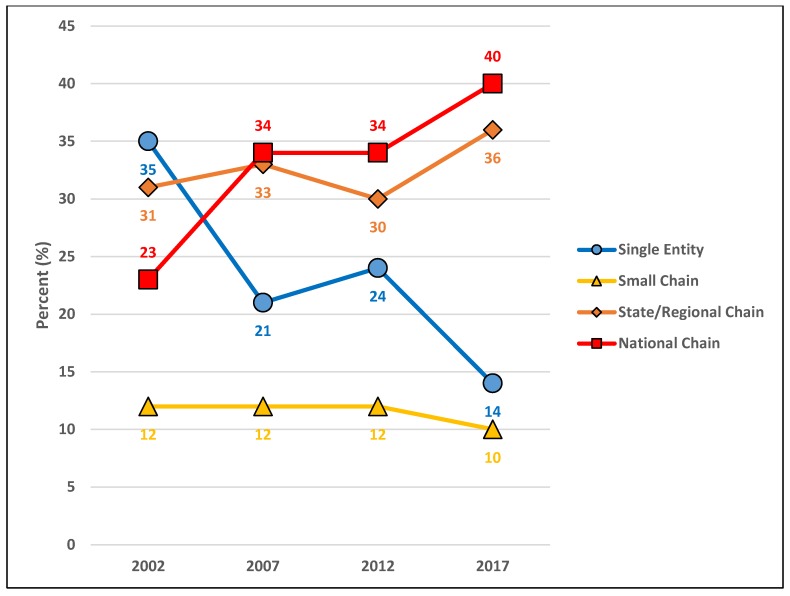
The proportion of business organization structures for community pharmacies in Minnesota for 2002, 2007, 2012, and 2017.

Figure 8 depicts the proportional makeup of community pharmacies in Minnesota by business organization structures for 2002, 2007, 2012, and 2017. ‘Single entity’ pharmacies decreased from 35% in 2002 to 21% in 2007, rose to 24% in 2012 and then fell to 14% in 2017. ‘Small chain’ pharmacies remained stable at 12% from 2002 through 2007 and 2012 but decreased to 10% in 2017. ‘State/regional chain’ pharmacies rose from 31% in 2002 to 33% in 2007, fell to 30% in 2012 and then rose 36% in 2017. ‘National chain’ community pharmacies rose from 23% in 2002 to 34% in 2007, remained stable at 34% in 2012 and then rose to 40% in 2017.

**Figure 9:**
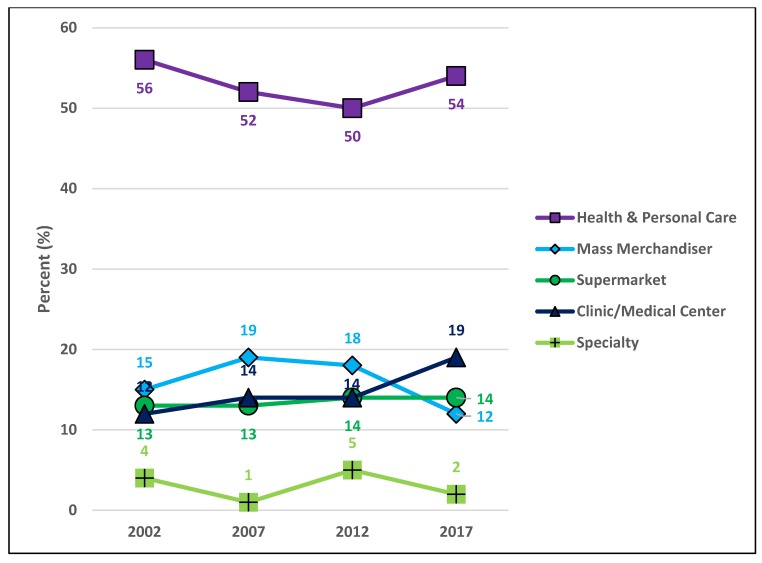
The proportion of community pharmacy types in Minnesota for 2002, 2007, 2012, and 2017.

Figure 9 depicts the proportional makeup of community pharmacies in Minnesota by health and personal care pharmacy types for 2002, 2007, 2012, and 2017. Health and personal care pharmacy types decreased from 56% of ‘All community pharmacies’ in 2002 to 52% in 2007 and 50% in 2012, but then rose to 54% in 2017. ‘Mass Merchandiser’ pharmacies grew from 15% in 2002 to 19% in 2007, but fell to 18% in 2012 and then 12% in 2017. ‘Supermarket’ pharmacies stayed stable at 13% from 2002 to 2007 and the rose to 14% in 2012 and 2017. ‘Clinic/Medical Center’ pharmacies rose from 12% in 2002 to 14% in 2007 and 2012 and then increased again to 19% in 2017. Finally, ‘Specialty’ pharmacies fell from 4% in 2002 to 1% in 2007, before rising up to 5% in 2012 and falling again to 2% in 2017.

## 4. Discussion

The first objective of this study was to describe the market dynamics of community pharmacies for each county in the state of Minnesota every five years over a 15 year period (i.e., 2002–2017).

The results show that the total number of community pharmacies grew from 2002–2007 (966 to 1070) and 2007–2012 (1070 to 1094) but declined from 2012–2017 (1094 to 1063) resulting in an overall net increase of 97 community pharmacies over 15 years (see Table 2). The bulk of this overall increase took place in only a handful of Minnesota counties as each sequential five year period saw smaller proportions of counties gaining pharmacies and larger proportions of counties losing pharmacies. Each successive 5 year interval analyzed saw a weakening in the explanatory power of the independent variables of population density (*p*-values: 0.002 < 0.052 < 0.349; see Appendix A Table A1, Table A2 and Table A3) and metropolitan designation (*p*-values: 0.083 < 0.111 < 0.323; see Appendix A Table A4, Table A5 and Table A6) for the market dynamics of ‘All community pharmacies.’ This result deviates from previous research on the topic and indicates the need for additional investigation into the potential causes.

The total number of ‘Independent community pharmacies’ fell between 2002–2007 (466 to 352), then rose between 2007–2012 (352 to 387), before falling again in 2012–2017 (387 to 256) resulting in a net decrease of 210 ‘Independent community pharmacies’ over 15 years (see Table 2). The only statistically significant relationships with the market dynamics of ‘Independent community pharmacies’ were population density for 2007–2012 (*p* = 0.005; see Appendix A Table A2) and metropolitan designation for 2002–2007 (*p* = 0.021; see Appendix A Table A4) suggesting the importance of factors beyond just population density and metropolitan designation are driving market dynamics.

The total number of ‘Chain community pharmacies’ was inversely related to ‘Independent community pharmacies’ displaying an increase between 2002–2007 (530 to 718), a decline between 2007–2012 (718 to 707), followed by growth again in 2012–2017 (707 to 807) resulting in a net increase of 277 ‘Chain community pharmacies’ over 15 years (see Table 2). Population density retained a statistically significant relationship with market dynamics of ‘Chain community pharmacies’ throughout each five year interval of the study (*p*-values: 0.014, 0.009, 0.022; see Appendix A Table A1, Table A2 and Table A3), while metropolitan designation was significant only for the five year interval between 2012–2017 (*p* = 0.013; see Appendix A Table A6). This again suggests that other factors beyond metropolitan designation would be useful for data interpretation.

When analyzed as a whole, the results do not yield a consistent relationship between the study’s independent variables and the market dynamic patterns that carry throughout the analyzed time periods. This finding differs from the conclusions of previous work by Schommer et al. that suggested the best opportunities for growth in the number of pharmacies occurred where population density was increasing and adequate infrastructure, logistics, resources, and markets, represented by a metropolitan area designation, existed [9]. This suggests that different or additional factors beyond the traditional market dynamic predictors were at play in each 5 year intervals of this study. We propose that the traditional dichotomy of independent and chain community pharmacy groupings no longer provide an optimal characterization for the market dynamics of pharmacies today. Each of these possibilities is better evaluated as a part of this study’s second objective. 

The second objective was to describe the number and proportion of community pharmacies for each county in the state of Minnesota every five years over a 15 year period (i.e., 2002–2017) by (1) ‘Business Organization Structure’ and (2) ‘Pharmacy Type.’ 

In 2002, ‘Single entity’—‘Independent community pharmacies’ represented the highest number and proportion of business organization structures for community pharmacies in the state of Minnesota (See Table 2). By 2007, these totals plummeted from 35% (N = 346) to 21% (N = 227), with gains by ‘State/regional chains’ and ‘National chains’ surpassing the overall difference (see Figure 8). Previous research has suggested this result as being the consequence of the latter outcompeting and acquiring the former in conveniently dispensing medications to patients utilizing a product-oriented, fee for service reimbursement systems [9]. These conditions combined with a strong economy may have also led to overall increases in ‘State/regional chain’ and ‘National chain’ community pharmacies between 2002–2007 that surpassed what would be expected based on population densities and metropolitan designation (See Table 2). Community pharmacy types between 2002–2007 remained fairly stable with the most notable changes being a four-point decline in ‘Health and personal care’ community pharmacies and a four-point rise in ‘Mass merchandiser’ pharmacies. The decrease in the former reflects losses of independent ‘Single entity’ community pharmacies explained at the beginning of the paragraph which was offset by gains of ‘State/regional chains’ in this category. The four-point rise of ‘Mass merchandiser’ community pharmacies also was accounted for by the growth of ‘National chains’ and reflected the expansion of big box stores like Walmart nationwide during this time period.

The interval between 2007–2012 saw a relative reversal of the business organization structure trends from the preceding five years, although not at the same magnitude as 2002–2007. ‘Single entity’—‘Independent community pharmacies’ grew in number (227 to 260) and proportion (21% to 24%), while ‘State/regional chains’ declined and ‘National chains’ held steady (see Figure 8). This significant change in market dynamic trends points to macro forces at play such as the near economic collapse of 2008 and passage of the Affordable Care Act of 2010 (ACA). It may be that these events created an uncertain and altered business environment difficult for larger healthcare entities to deftly navigate, which created niche opportunities for the more nimble ‘Independent community pharmacies’ to fill. In some cases, pharmacists who left chain positions may have utilized their experiential knowledge to start new businesses in areas such as compounding, home infusion, and other specialty services that were historically not thought of or classified as independent community pharmacies. Additionally, the ACA was a major piece of healthcare legislation that may represent a watershed moment for community pharmacy practice and market dynamics by increasing the business viability of pharmacies as healthcare access points with the capacity to provide and be reimbursed for patient care and public health services like medication therapy management, immunizations, and more. Community pharmacy types between 2007–2012 continued to remain fairly stable with small declines in ‘Health and personal care’ pharmacies and ‘Mass merchandiser’ community pharmacies with the difference made up by small increases in ‘Specialty’ community pharmacies.

The market dynamic trends of business organization structure changed again between 2012–2017, as ‘Single entity’—independent community pharmacies saw a return of sharp declines in their number and proportion (see Figure 8 and Table 2) and large rises in ‘State/regional chains’ and ‘National chains.’ These findings suggest that these larger community pharmacy entities adjusted to the new healthcare law and were bolstered by a stronger economy to once again outpace the growth of ‘Single entity’ and ‘Small chain’ community pharmacies through by beginning to providing patient care services along with its convenient dispensing. This time period also saw chain pharmacies grow or attempt to grow via mergers and acquisitions for the purposes of horizontal integration (e.g., CVS acquiring Target pharmacies, Walgreens acquiring Rite Aid) to increase customer access and vertical integration (e.g., CVS-Aetna, Walgreens-AmerisourceBergen, Walmart-Humana) to contain customers within comprehensive service networks. This is particularly evidenced by the type of community pharmacies that showed the most growth during this time period. ‘Clinic/medical center’ community pharmacies showed the greatest increase in number and proportion, indicating their natural advantage over other pharmacy types in integrating advanced pharmacist services and patient information into comprehensive and quality healthcare services. The time periods between 2012–2017 also showed a rise in ‘Health and personal care’ community pharmacies like CVS, Thrifty White, and Walgreens which also began offering patient care services. The largest decline during this time period was ‘Mass merchandiser’ community pharmacies, which reflected a reduction in the number of box stores nationwide due to a general shift in consumer preferences for online retail providers like Amazon [25].

Another interesting note pertaining to market dynamics is the limited growth of ‘Specialty’ community pharmacies in comparison to the proportional growth of healthcare dollars on specialty drugs over the 15 year period of the study. In fact, even the small fluctuations in ‘Specialty’ pharmacies may be due to methodological variation from how the category was defined/coded at the time of this study (2007 and 2017 data) and the previous work by Schommer et al. that this study drew from (2002 and 2012 data).

### Study Limitations

The limitations of this study should be considered when interpreting the findings. First, these analyses were performed for only 87 counties making up a single state and therefore did not account for the outside influences or characteristics of border counties that could impact affect market dynamics, making multivariate statistical analysis impractical. Another factor that was not accounted for was the impact of insurance mandates for prescription mail order services or changes to third party payment contracts, particularly for independent/small chain pharmacies. Furthermore, the limited number of counties prevented the use of multivariate statistical analysis. Another limitation of the study is that only licensed community pharmacies were considered, rather than all locations where pharmacist services are provided such as in managed care organizations and medical centers. The inclusion of these entities would be outside the scope of this paper, but future research focusing on the type and quality of pharmacist services rather than the business structure and location of pharmacies. Finally, there may be other characteristics pertaining to pharmacy organizations and the demographics they serve which can explain the market dynamics in this study.

## 5. Conclusions

The findings showed that community pharmacy distribution in Minnesota’s 87 counties may have shifted between 2002 and 2017 from traditional retail models to emerging healthcare models based on population health needs. This signals the need for not only a new approach for tracking community pharmacy market dynamics but also adjustments by community pharmacies to remain relevant in a new environment of patient care services. 

## Figures and Tables

**Table 1:** Market dynamics for community pharmacies in Minnesota counties every five years between 2002 and 2017 (N = 87).

Pharmacy Category Market Dynamic	2002–2007	2007–2012	2012–2017
All community pharmacies			
Lost pharmacies	18%	29%	38%
Stayed the same	36%	43%	40%
Gained pharmacies	46%	29%	22%
Independent pharmacies			
Lost pharmacies	55%	33%	54%
Stayed the same	25%	29%	30%
Gained pharmacies	20%	38%	16%
Chain pharmacies			
Lost pharmacies	8%	31%	16%
Stayed the same	36%	38%	45%
Gained pharmacies	56%	31%	39%

Notes: percentages may not total 100% due to rounding.

**Table 2:** Community pharmacy business organization structures and pharmacy types in Minnesota for 2002, 2007, 2012, 2017 (Number, column %).

Business Organization Structure	Pharmacy Type	2002	2007	2012	2017
		N = 996	N = 1070	N = 1094	N = 1063
Single entity	Health & Personal Care	281 (28%)	185 (17%)	199 (18%)	127 (12%)
	Mass merchandiser	0 (0%)	0 (0%)	0 (0%)	0 (0%)
	Supermarket	0 (0%)	0 (0%)	0 (0%)	0 (0%)
	Clinic/medical center	36 (4%)	32 (3%)	28 (3%)	19 (2%)
	Specialty	29 (3%)	10 (1%)	33 (3%)	5 (1%)
Total		346 (35%)	227 (21%)	260 (24%)	151 (14%)
Small chain	Health & Personal Care	98 (10%)	97 (9%)	91 (8%)	83 (8%)
	Mass merchandiser	0 (0%)	0 (0%)	0 (0%)	0 (0%)
	Supermarket	0 (0%)	0 (0%)	0 (0%)	0 (0%)
	Clinic/medical center	20 (2%)	26 (2%)	30 (3%)	17 (2%)
	Specialty	2 (<1%)	2 (<1%)	6 (1%)	5 (1%)
Total		120 (12%)	125 (12%)	127 (12%)	105 (10%)
ALL INDEPENDENTS (=Single entity +Small chain)		466 (47%)	352 (33%)	387 (35%)	256 (24%)
State/regional	Health & Personal Care	99 (10%)	120 (11%)	63 (6%)	66 (6%)
chain	Mass merchandiser	13 (1%)	0 (0%)	17 (2%)	0 (0%)
	Supermarket	127 (13%)	139 (13%)	155 (14%)	150 (14%)
	Clinic/medical center	61 (6%)	96 (9%)	83 (8%)	162 (15%)
	Specialty	5 (1%)	0 (0%)	12 (1%)	5 (1%)
Total		305 (31%)	355 (33%)	330 (30%)	383 (36%)
National chain	Health & Personal Care	81 (8%)	152 (14%)	201 (18%)	294 (28%)
	Mass merchandiser	144 (14%)	209 (20%)	173 (16%)	127 (12%)
	Supermarket	0 (0%)	0 (0%)	0 (0%)	0 (0%)
	Clinic/medical center	0 (0%)	0 (0%)	0 (0%)	0 (0%)
	Specialty	0 (0%)	2 (<1%)	3 (<1%)	2 (<1%)
Total		225 (23%)	363 (34%)	377 (34%)	423 (40%)
ALL CHAIN (=State/regional chain + National chain)		530 (53%)	718 (67%)	707 (65%)	807 (76%)

Notes: percentages may not total 100% due to rounding.

## Appendix A

**Table A1:** The relationship between change in population density (p/mi^2^) and market dynamics for community pharmacies in Minnesota counties between 2002 and 2007 (N = 87).

Pharmacy Category Market Dynamic	Counties with <0 p/mi^2^ Change	Counties with 0–5 p/mi^2^ Change	Counties with >5 p/mi^2^ Change	Overall
	N = 43	N = 29	N = 15	N = 87
All community pharmacies				
Lost pharmacies	23%	17%	7%	18%
Stayed the same	51%	24%	13%	36%
Gained pharmacies	26%	59%	80%	46%
*p* = 0.002				
Independent pharmacies				
Lost pharmacies	56%	41%	80%	55%
Stayed the same	23%	35%	13%	25%
Gained pharmacies	21%	24%	7%	20%
*p* = 0.185				
Chain pharmacies				
Lost pharmacies	12%	3%	7%	8%
Stayed the same	42%	45%	0%	36%
Gained pharmacies	47%	52%	93%	56%
*p* = 0.014				

Notes: p/mi^2^ = persons per square mile; percentages may not total 100% due to rounding.

**Table A2:** The relationship between change in population density (p/mi^2^) and market dynamics for community pharmacies in Minnesota counties between 2007 and 2012 (N = 87).

Pharmacy Category Market Dynamic	Counties with <0 p/mi^2^ Change	Counties with 0–5 p/mi^2^ Change	Counties with >5 p/mi^2^ Change	Overall
	N = 44	N = 31	N = 12	N = 87
All community pharmacies				
Lost pharmacies	30%	26%	33%	29%
Stayed the same	55%	36%	17%	43%
Gained pharmacies	16%	39%	50%	29%
*p* = 0.052				
Independent pharmacies				
Lost pharmacies	43%	23%	25%	33%
Stayed the same	39%	19%	17%	29%
Gained pharmacies	18%	58%	58%	38%
*p* = 0.005				
Chain pharmacies				
Lost pharmacies	18%	45%	42%	31%
Stayed the same	54%	26%	8%	38%
Gained pharmacies	27%	29%	50%	31%
*p* = 0.009				

Notes: p/mi^2^ = persons per square mile; percentages may not total 100% due to rounding.

**Table A3:** The relationship between change in population density (p/mi^2^) and market dynamics for community pharmacies in Minnesota counties between 2012 and 2017 (N = 87).

Pharmacy CategoryMarket Dynamic	Counties with <0 p/mi^2^ Change	Counties with 0–5 p/mi^2^ Change	Counties with >5 p/mi^2^ Change	Overall
	N = 44	N = 33	N = 10	N = 87
All community pharmacies				
Lost pharmacies	32%	46%	40%	38%
Stayed the same	48%	36%	20%	40%
Gained pharmacies	21%	18%	40%	22%
*p* = 0.349				
Independent pharmacies				
Lost pharmacies	43%	61%	80%	54%
Stayed the same	36%	27%	10%	30%
Gained pharmacies	21%	12%	10%	16%
*p* = 0.234				
Chain pharmacies				
Lost pharmacies	16%	12%	30%	16%
Stayed the same	59%	36%	10%	45%
Gained pharmacies	25%	52%	60%	39%
*p* = 0.022				

Notes: p/mi^2^ = persons per square mile; percentages may not total 100% due to rounding.

**Table A4:** The relationship between Metropolitan Designation and market dynamics for community pharmacies in Minnesota counties between 2002 and 2007 (N = 87).

Pharmacy CategoryMarket Dynamic	Metropolitan Counties	Non-Metropolitan Counties	Overall
	N = 21	N = 66	N = 87
All community pharmacies			
Lost pharmacies	14%	19%	18%
Stayed the same	19%	41%	36%
Gained pharmacies	67%	39%	46%
*p* = 0.083			
Independent pharmacies			
Lost pharmacies	81%	47%	55%
Stayed the same	14%	29%	25%
Gained pharmacies	5%	24%	20%
*p* = 0.021			
Chain pharmacies			
Lost pharmacies	5%	9%	8%
Stayed the same	19%	41%	36%
Gained pharmacies	76%	50%	56%
*p* = 0.108			

Notes: percentages may not total 100% due to rounding.

**Table A5:** The relationship between Metropolitan Designation and market dynamics for community pharmacies in Minnesota counties between 2007 and 2012 (N = 87).

Pharmacy CategoryMarket Dynamic	Metropolitan Counties	Non-Metropolitan Counties	Overall
	N = 21	N = 66	N = 87
All community pharmacies			
Lost pharmacies	33%	27%	29%
Stayed the same	24%	49%	43%
Gained pharmacies	43%	24%	29%
*p* = 0.111			
Independent pharmacies			
Lost pharmacies	33%	33%	33%
Stayed the same	14%	33%	29%
Gained pharmacies	52%	33%	38%
*p* = 0.171			
Chain pharmacies			
Lost pharmacies	48%	26%	31%
Stayed the same	24%	42%	38%
Gained pharmacies	29%	32%	31%
*p* = 0.138			

Notes: percentages may not total 100% due to rounding.

**Table A6:** The relationship between Metropolitan Designation and market dynamics for community pharmacies in Minnesota counties between 2012 and 2017 (N = 87).

Pharmacy CategoryMarket Dynamic	Metropolitan Counties	Non-Metropolitan Counties	Overall
	N = 27	N = 60	N = 87
All community pharmacies			
Lost pharmacies	41%	37%	38%
Stayed the same	30%	45%	40%
Gained pharmacies	30%	18%	22%
*p* = 0.323			
Independent pharmacies			
Lost pharmacies	67%	48%	54%
Stayed the same	22%	33%	30%
Gained pharmacies	11%	18%	16%
*p* = 0.282			
Chain pharmacies			
Lost pharmacies	19%	15%	16%
Stayed the same	22%	55%	45%
Gained pharmacies	59%	30%	39%
*p* = 0.013			

Notes: percentages may not total 100% due to rounding.
